# Side‐to‐side repair improves coronal meniscal extrusion in lateral meniscus oblique radial tears concomitant with anterior cruciate ligament reconstruction

**DOI:** 10.1002/jeo2.70916

**Published:** 2026-09-21

**Authors:** Kyota Ishibashi, Hikaru Kristi Ishibashi, Eiji Sasaki, Yuka Kimura, Yasuyuki Ishibashi

**Affiliations:** ^1^ Department of Orthopaedic Surgery Hirosaki University Graduate Schoo Hirosaki Japan

**Keywords:** anterior cruciate ligament reconstruction, lateral meniscus tear, magnetic resonance imaging, meniscal extrusion, meniscal repair

## Abstract

**Purpose:**

To determine the proportion of lateral meniscus oblique radial tear repairs during anterior cruciate ligament reconstruction and compare extrusion and clinical outcomes between side‐to‐side repair of lateral meniscus oblique radial tear and posterior longitudinal tear repair.

**Methods:**

Among 609 primary anterior cruciate ligament reconstructions from 2016 to 2023, 34 patients with intraoperatively confirmed lateral meniscus oblique radial tear underwent side‐to‐side repair and magnetic resonance imaging at 6 months and 1 year; 27 with posterior longitudinal tears underwent all‐inside repair. Coronal and sagittal lateral meniscal extrusion, clinical outcomes, knee stability and Knee Injury and Osteoarthritis Outcome Score subscales were compared. Multivariate regression assessed predictors of preoperative‐to‐1‐year coronal extrusion change; receiver operating characteristic analysis assessed whether preoperative lateral meniscal extrusion distinguished tear types.

**Results:**

Lateral meniscus oblique radial tear treated with side‐to‐side repair accounted for 7.4% of cases. Preoperative coronal lateral meniscal extrusion was greater in the lateral meniscus oblique radial tear group than in the longitudinal tear group (1.71 ± 1.31 vs. 0.32 ± 0.61 mm; *p* < 0.001). Coronal extrusion improved by approximately 0.6 mm after lateral meniscus oblique radial tear repair and was similar between groups at 1 year; sagittal extrusion increased slightly in both groups. Clinical outcomes were similar at 1 year and final follow‐up. Preoperative coronal lateral meniscal extrusion was an independent predictor of coronal extrusion change (*β* = −0.514, *p* < 0.001). Preoperative coronal lateral meniscal extrusion >1.0 mm identified lateral meniscus oblique radial tear with acceptable diagnostic performance (area under the curve = 0.787, *p* < 0.001).

**Conclusions:**

Side‐to‐side lateral meniscus oblique radial tear repair during anterior cruciate ligament reconstruction reduced coronal extrusion by 1 year to levels comparable to those of longitudinal tear repair, with similar clinical outcomes. These findings support side‐to‐side repair as a reliable meniscal preservation strategy.

**Level of Evidence:**

Level III.

**Abbreviations**
ACL: anterior cruciate ligament
ACLR: anterior cruciate ligament reconstruction
ADL: activities of daily living
ADT: anterior drawer test
ANOVA: analysis of variance
AUC: area under curve
BMI: body mass index
CI: confidence interval
KOOS: Knee injury and Osteoarthritis Outcome Score
LM: lateral meniscus
LME: lateral meniscal extrusion
LMORT: lateral meniscal oblique radial tear
MM: medial meniscus
MRI: magnetic resonance imaging
QOL: quality of life
ROC: receiver operating characteristic

## INTRODUCTION

Lateral meniscus (LM) injury is frequently observed in patients with anterior cruciate ligament (ACL) injury, with more than half of acute cases involving some degree of meniscal damage [3, 15, 25]. Among these injuries, the lateral meniscus oblique radial tear (LMORT) pattern has gained attention because disruption of the circumferential fibres compromises hoop stress transmission and may increase rotational instability and compartmental contact pressure [13]. These biomechanical alterations may persist even after anatomic ACL reconstruction (ACLR) if the lesion remains unrepaired [21].

Although magnetic resonance imaging (MRI) is useful for evaluating meniscal morphology, differentiating LMORT from posterior longitudinal tears in clinical practice can be challenging because both lesions often demonstrate similar signal patterns. MRI can differentiate LMORT from other posterior LM tears with 77% sensitivity and 55% specificity [1]. Meniscal extrusion is an important imaging finding because increased extrusion reflects loss of hoop tension and has been associated with progressive cartilage degeneration and the subsequent development of knee osteoarthritis [14, 18]. However, preoperative MRI features that may help surgeons identify LMORT before arthroscopy have not been systematically characterised, and postoperative changes in LMORT after repair remain poorly understood.

Side‐to‐side repair has been proposed to restore continuity of the disrupted circumferential fibres in LMORT, and recent cadaveric studies have demonstrated that this technique can restore hoop tension and reduce abnormal contact pressure to near‐intact levels [21]. However, clinical evidence supporting the in vivo efficacy of side‐to‐side repair for LMORT remains limited, and few studies have incorporated sequential MRI evaluations. Most previous clinical investigations have focused on symptomatic improvement, postoperative knee stability or second‐look arthroscopy, whereas it remains unclear whether this technique can effectively reduce postoperative meniscal extrusion and yield clinical outcomes comparable to those achieved after repair of more commonly encountered tear patterns, such as posterior longitudinal tears. Addressing these gaps is clinically important for guiding surgical decision‐making and optimising meniscal preservation in patients with ACL injury.

The aims of this study were threefold: (1) to determine the proportion of LMORT repairs among patients undergoing primary ACLR, (2) to evaluate postoperative meniscal extrusion and mid‐term clinical outcomes after side‐to‐side repair of LMORT and (3) to compare preoperative MRI characteristics between LMORT and posterior longitudinal LM tears. It was hypothesised that (1) LMORT would account for a clinically relevant proportion of meniscal repairs performed during primary ACLR, (2) side‐to‐side repair of LMORT would result in favourable clinical outcomes and postoperative meniscal extrusion comparable to that after repair of posterior longitudinal tears and (3) preoperative coronal meniscal extrusion would be greater in LMORT than in posterior longitudinal tears, thereby serving as a potential diagnostic indicator on preoperative MRI.

## METHODS

### Study design and population

This retrospective study was approved by the ethics committee of Hirosaki University (2025‐073) and conducted in accordance with the 1964 Declaration of Helsinki and subsequent amendments. All participants provided informed consent before enrolment. Clinical data were reviewed for patients who underwent ACLR at Hirosaki University between 2016 and 2023. During this period, 719 ACLRs were performed for ACL injury diagnosed by clinical examination and MRI.

**Figure 1:**
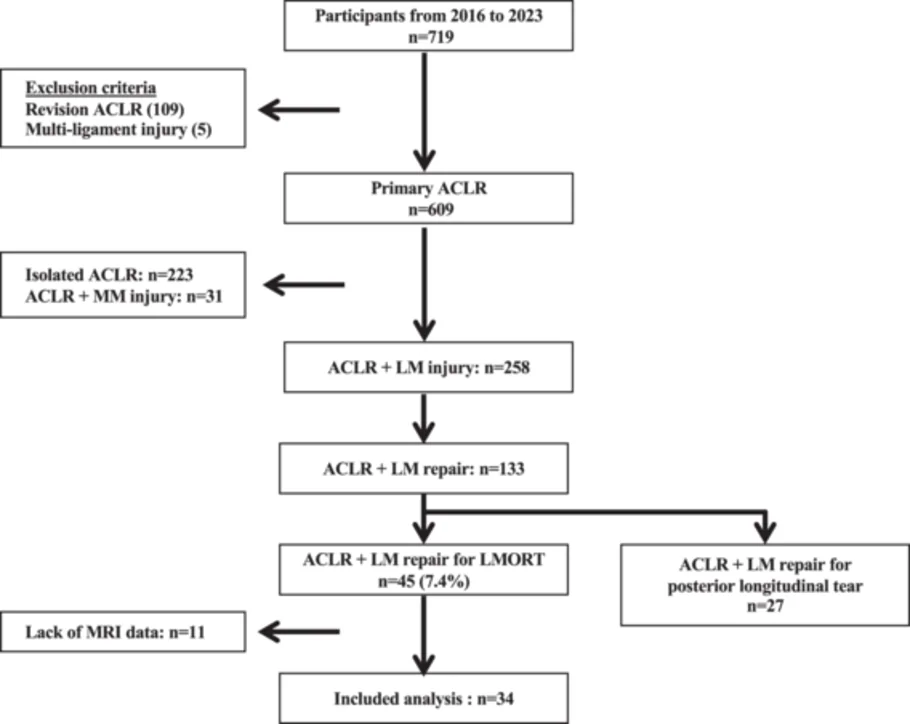
Flowchart of patient selection. Flowchart showing the identification of eligible patients from 2016 to 2023. ACLR, anterior cruciate ligament reconstruction; LM, lateral meniscus; LMORT, lateral meniscus oblique radial tear; MM, medial meniscus; MRI, magnetic resonance imaging.

Inclusion criteria were (1) primary anatomic double‐bundle ACLR using a hamstring tendon autograft; (2) intraoperatively confirmed LMORT or posterior horn longitudinal LM tear treated with repair; (3) complete clinical records and preoperative, 6‐month, and 1‐year postoperative MRI data and (4) at least 2 years of postoperative follow‐up. Exclusion criteria were (1) revision ACLR; (2) concomitant multiligament injuries; (3) ACL injury managed without reconstruction; and (4) incomplete required MRI follow‐up. After applying these criteria, 609 patients who underwent primary ACLR were eligible. Patients with intraoperatively confirmed LMORT treated by side‐to‐side repair formed the LMORT group. The comparison group included patients with repaired posterior horn longitudinal LM tears and complete clinical and MRI data (Figure 1).

Time‐dependent LM changes were assessed using preoperative, 6‐month and 1‐year postoperative MRI.

### Surgical technique

**Figure 2:**
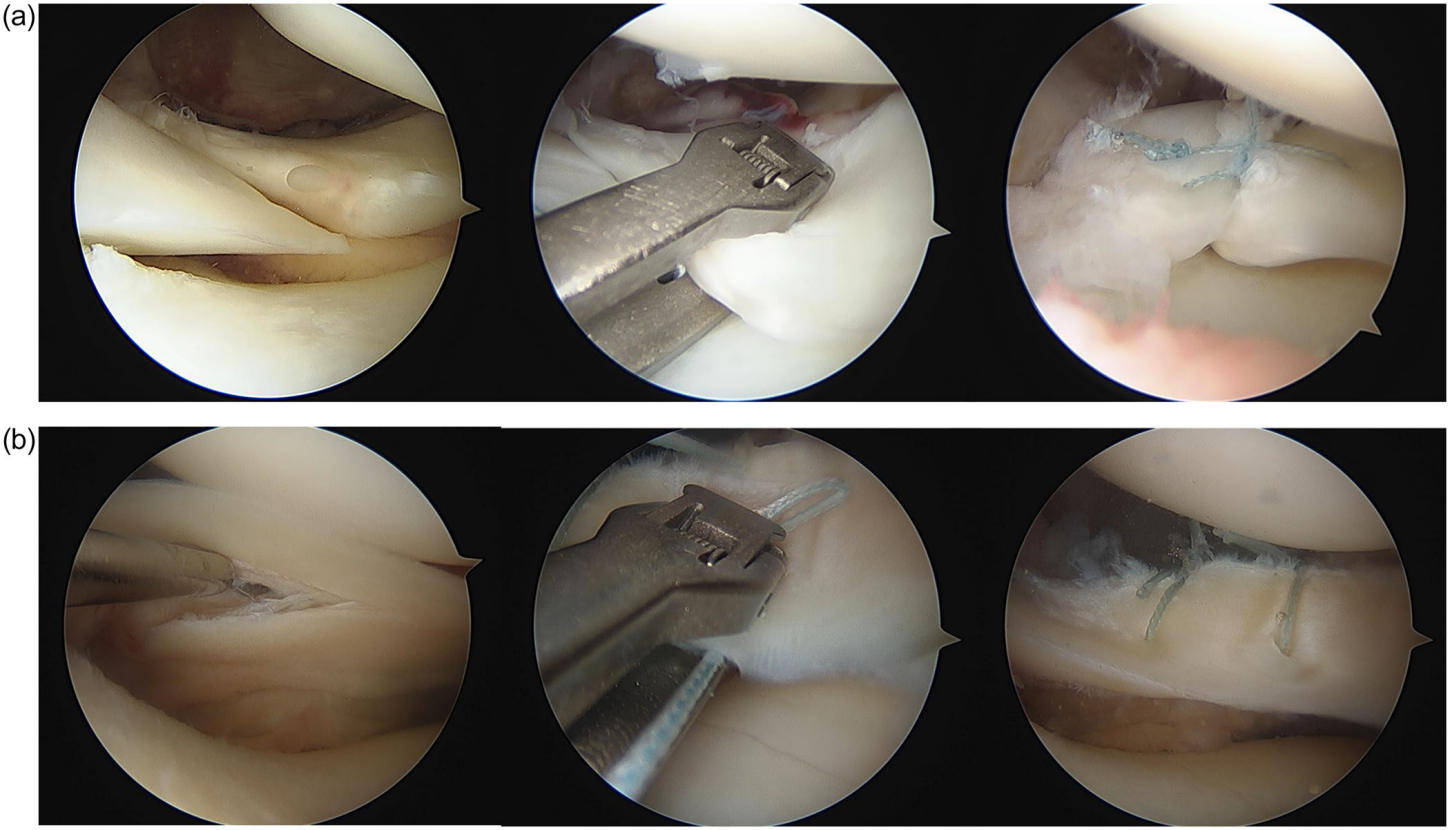
Arthroscopic findings of lateral meniscus oblique radial tear (LMORT) and longitudinal tears. (a) LMORT of the posterior horn of the lateral meniscus. Left: oblique radial disruption of the circumferential fibres. Middle: side‐to‐side repair using a suture passer. Right: appearance after side‐to‐side repair. (b) Posterior longitudinal tear of the lateral meniscus. Left: longitudinal tear of the posterior horn. Middle: all‐inside repair using a suture passer. Right: appearance after all‐inside repair. Arthroscopic images were obtained with the knee in the figure‐of‐four position under fluid distension, which opens the lateral compartment; therefore, they do not represent resting meniscal position. Extrusion was measured on MRI. MRI, magnetic resonance imaging.

All patients underwent anatomic double‐bundle ACLR using hamstring tendon grafts as previously described [11, 16, 20]. After diagnostic arthroscopy, the menisci were systematically inspected through standard anterolateral and anteromedial portals. Tear morphology and location were assessed, including longitudinal, oblique radial and complex patterns. LMORT was defined as an oblique radial tear of the posterior horn of the LM and classified as Types 1 to 4 according to severity and distance from the meniscal root attachment [17]. Types 3 and 4 represent partial and complete oblique radial tears extending more than 10 mm from the root and have the greatest effect on knee stability and meniscal function. Side‐to‐side repair was performed when the torn meniscal fragment was readily retractable with probing or continuous bridging fibres were absent. Posterior longitudinal LM tears were repaired with vertical sutures (Figure 2). Stable tears <10 mm in size or those with intact tissue continuity were left in situ because short, stable lateral meniscal tears in ACL‐injured knees may remain asymptomatic with a low rate of subsequent meniscal surgery, whereas unnecessary suture passage may compromise peripheral meniscal vascularity [15]. All repairs used an all‐inside technique with a Knee Scorpion suture passer (Arthrex).

The semitendinosus tendon was harvested and prepared as two separate grafts for the anteromedial and posterolateral bundles. The gracilis tendon was harvested as needed to increase graft diameter. Femoral fixation used an adjustable‐loop cortical suspensory device (TightRope, Arthrex) to provide adequate graft length within the femoral tunnel and maximise the graft–bone interface area. Each bundle was fixed independently on the tibial side using TensionLoc (Arthrex), with maximal manual tension applied at 15°–20° of knee flexion.

### Postoperative rehabilitation

Rehabilitation followed a standardised protocol. Weight‐bearing with crutches was permitted the day after surgery. After meniscal repair, the knee was immobilised in extension with a brace for approximately 1 week. Progressive range‐of‐motion and closed kinetic chain exercises were initiated early, followed by running, open kinetic chain strengthening, and jump–landing training at approximately 3 months. Return to preinjury sports was permitted between 6 and 9 months based on quadriceps strength, neuromuscular control and functional stability. Return to pivoting and contact sports required an isokinetic quadriceps limb symmetry index of at least 80%, a negative pivot‐shift test, and completion of a supervised jump–landing program.

### Radiographic measurements

Radiographic assessments were performed using standard anteroposterior weight‐bearing radiographs. Lateral joint space width was measured as the distance between the centre of the lateral femoral condyle and the centre of the lateral tibial plateau. Measurements were obtained 1 year postoperatively and at final follow‐up using Picture Archiving and Communication System software (ShadeQuest/ViewR, version 1.24.15; Fujifilm Medical Co., Ltd.), with measurement precision of 0.01° for angles and 0.01 mm for lengths.

### MRI measurements

MRI examinations were performed using a 1.5 Tesla scanner (Signa HDxt; GE Healthcare). At each assessment, the knee was immobilised in a custom frame at approximately 10° of flexion with 10°–15° of external rotation to standardise visualisation of the LM. Sagittal fast spin‐echo proton density‐weighted and T2‐weighted images were obtained. For the proton density‐weighted sequence, the repetition time was 3000 ms, echo time 16 ms, slice thickness 4.0 mm with a 1.0 mm interslice gap, field of view 16 cm, two excitations, echo train length 14, and bandwidth 631.25 kHz per field of view. The T2‐weighted sequence used the same slice parameters, with a repetition time of 3000 ms and an echo time of 98 ms.

**Figure 3:**
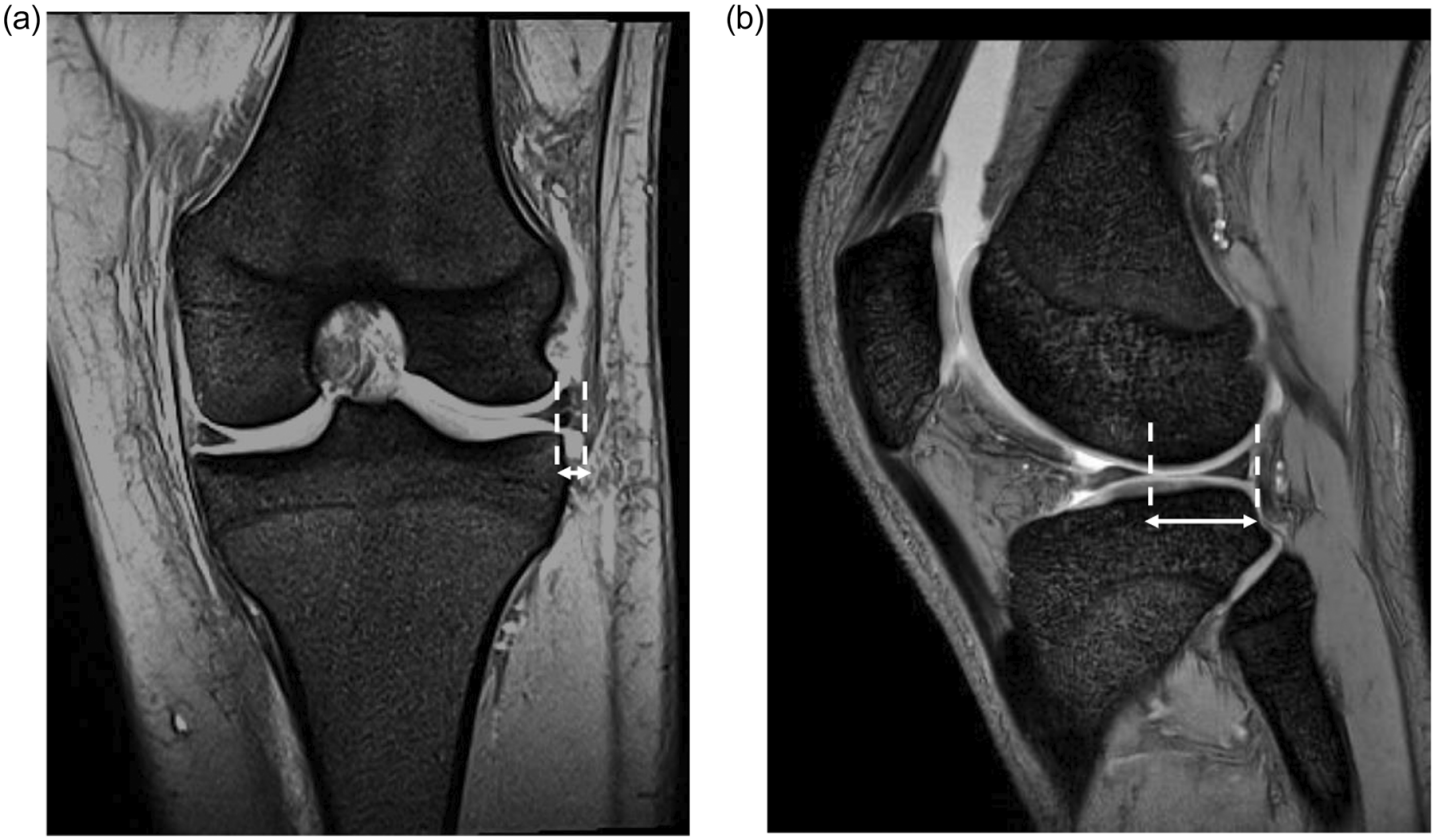
Measurement of lateral meniscal extrusion on MRI. (a) Coronal image showing measurement of coronal lateral meniscal extrusion. (b) Sagittal image showing measurement of sagittal lateral meniscal extrusion. MRI, magnetic resonance imaging.

Lateral meniscal extrusion (LME) was measured in the coronal and sagittal planes as previously described [23]. Coronal LME was defined as the horizontal distance from the outer margin of the lateral tibial plateau to the meniscocapsular junction on the midcoronal slice. Sagittal LME was defined as the distance between the inner margin of the anterior portion of the LM and the meniscocapsular junction of its posterior portion on the midsagittal slice (Figure 3).

### Clinical evaluation

Demographic variables, including age, sex, body mass index (BMI) and Tegner activity scale, were recorded for all patients. Pre and postoperative assessments included knee stability testing with side‐to‐side differences in anterior tibial translation measured using a KT‐1000 arthrometer (MEDmetric Corp.), as well as pivot‐shift, anterior drawer, and Lachman tests. A pivot‐shift grade of one or higher was considered positive. Two senior orthopaedic surgeons, each with more than 20 years of clinical experience, performed all postoperative examinations. Revision ACLR and meniscal repair‐related reoperations were recorded during follow‐up.

Patient‐reported outcomes were assessed using the knee injury and osteoarthritis outcome score (KOOS) at 1 year and final follow‐up [2, 19]. KOOS subscales included symptoms, pain, activities of daily living, sports and quality of life.

### Statistical analysis

Continuous variables are presented as means and standard deviations. Normality was assessed using the Shapiro–Wilk test. Because several continuous variables did not follow a normal distribution, the LMORT and longitudinal tear groups were compared using the Mann–Whitney U‐test. Categorical variables were compared using the chi‐square or Fisher's exact test. Changes in KOOS subscale scores and meniscal extrusion over time (at 6 months and at 1 year postoperatively) were evaluated using one‐way analysis of variance, followed by Bonferroni‐adjusted post hoc pairwise comparisons.

Multiple linear regression was used to identify factors associated with changes in LME from preoperative assessment to 1 year postoperatively. Changes in coronal or sagittal LME were the dependent variables, and age, sex, BMI, LMORT, number of repair sutures and preoperative LME were independent variables. A post hoc sample size calculation for the regression model was based on the observed effect size. With seven independent variables, *α* = 0.05, statistical power of 0.80, and an observed Cohen's *f* ^2^ of 0.93 (*R* ^2^ = 0.48), the minimum required sample size was 23 patients [G*Power]. Receiver operating characteristic (ROC) analysis evaluated the ability of preoperative coronal and sagittal LME to distinguish LMORT from posterior horn longitudinal tears. The area under the curve (AUC) quantified diagnostic performance, and the optimal cutoff was defined as the point closest to the upper left corner of the ROC curve. Statistical significance was defined as *p* < 0.05. Analyses were performed using SPSS version 29.0 (IBM Corp.).

## RESULTS

Of 609 primary ACLRs, 45 knees (7.4%) had intraoperatively confirmed LMORT treated with side‐to‐side repair. After excluding 11 with incomplete MRI data, 34 patients comprised the LMORT group; 27 comprised the longitudinal tear group. One LMORT was Type 4 and the remaining 33 were Type 3. Mean follow‐up was 32.5 ± 16.4 months (range, 24–96 months) for the overall cohort. The 61‐patient cohort exceeded the minimum sample size of 23 derived from the post hoc power calculation.

The groups did not differ significantly in age (25.2 ± 13.5 vs. 22.1 ± 8.9 years, *p* = 0.306), BMI (23.6 ± 3.9 vs. 24.4 ± 2.9 kg/m^2^, *p* = 0.405), sex distribution (16 [59%] vs. 20 [59%] female, *p* = 0.987), Tegner activity scale (*p* = 0.857), or follow‐up duration (*p* = 0.940) (Table 1). The frequencies of concomitant medial meniscus repair (*p* = 0.605), microfracture (*p* = 1.000), and free‐body removal (*p* = 1.000) also did not differ between groups (Table 2). At 1 year, knee stability did not differ significantly between groups based on the KT‐1000 side‐to‐side difference (*p* = 0.697), pivot‐shift test (*p* = 0.707) or Lachman test (*p* = 1.000). At final follow‐up, however, the KT‐1000 side‐to‐side difference was significantly smaller in the LMORT group than in the longitudinal tear group (0.3 ± 0.1 mm vs. 0.7 ± 0.2 mm, *p* = 0.036) (Table 3). Revision surgery occurred in three patients (10%) in the LMORT group and three patients (11%) in the longitudinal tear group (*p* = 0.975). All revisions were for ACL graft re‐rupture; none were performed for meniscal repair failure.

**Table 1:** Patients’ demographic data by group.

	Longitudinal	LMORT	*p*‐value
Number	27	34	
Age (years)	25.2 ± 13.5	22.1 ± 8.9	0.306
BMI (kg/m^2^)	23.6 ± 3.9	24.4 ± 2.9	0.405
Sex (female, *n* [%])	16 (59%)	20 (59%)	0.987
Tegner activity scale (range)	6.3 (1–9)	6.3 (2–8)	0.857
Follow‐up (months)	32.3 ± 16.4	33.6 ± 16.4	0.940
Sports			
Basketball	8	12	
Ski/snowboard	4	4	
Volleyball	1	5	
Soccer	2	3	
Badminton	2	2	
Gymnastics	0	2	
Baseball	1	0	
Handball	1	0	
Rugby	1	0	
Sumo	1	0	
Swimming	1	0	
Tennis	1	0	
Track and field	1	1	
None	3	5	

*Note*: Mean ± standard deviations of demographic data are shown. Abbreviations: BMI, body mass index; LMORT, lateral meniscus oblique radial tear; *n*, number. *p*‐values are exact values; the distribution of sports activities is presented as descriptive statistics only, and no statistical comparison was performed for this item.

**Table 2:** Concomitant procedures by group.

	Longitudinal (%)	LMORT (%)	*p*‐value
Medial meniscus repair	13 (48)	13 (38)	0.605
Microfracture	0 (0)	1 (3)	1.000
Free‐body removal	0 (0)	1 (3)	1.000

*Note*: Data are reported as *n* (%). LMORT, lateral meniscus oblique radial tear.

**Table 3:** Postoperative clinical and radiographic outcomes.

	Longitudinal	LMORT	*p*‐value
Revision (%)	3 (11)	3 (10)	0.975
1‐year follow‐up			
Lateral joint space (mm)	5.1 ± 0.7	5.2 ± 0.8	0.362
KT‐1000 side‐to‐side difference (mm)	0.6 ± 0.2	0.5 ± 0.2	0.697
Pivot‐shift test	5 (19)	4 (12)	0.707
ADT	2 (7)	2 (6)	1.000
Lachman test	2 (7)	3 (8)	1.000
KOOS symptom	89.6 ± 10.9	89.0 ± 10.7	0.836
KOOS pain	96.8 ± 5.8	93.5 ± 10.8	0.128
KOOS QOL	86.1 ± 17.7	87.3 ± 17.6	0.795
KOOS ADL	98.4 ± 3.8	96.9 ± 6.2	0.279
KOOS sports	82.0 ± 17.9	76.1 ± 21.4	0.255
Final follow‐up			
Lateral joint space (mm)	4.9 ± 0.6	5.1 ± 0.8	0.492
KT‐1000 side‐to‐side difference (mm)	0.7 ± 0.2	0.3 ± 0.1	0.036
Pivot‐shift test	3 (11)	5 (15)	0.975
ADT	1 (4)	1 (3)	1.000
Lachman test	1 (4)	1 (3)	1.000
KOOS symptoms	94.9 ± 10.4	91.6 ± 9.9	0.221
KOOS pain	97.8 ± 4.6	95.3 ± 6.7	0.129
KOOS QOL	90.7 ± 16.7	87.5 ± 15.5	0.439
KOOS ADL	98.8 ± 4.0	99.0 ± 2.4	0.855
KOOS sports	94.4 ± 9.2	92.4 ± 14.5	0.496

*Note*: Values are presented as mean ± standard deviation or number (%). Knee stability was assessed at 1 year and final follow‐up using the KT‐1000 side‐to‐side difference, pivot‐shift test, anterior drawer test (ADT), and Lachman test. Lateral joint space width and KOOS subscale scores for symptoms, pain, ADL, sports and QOL were compared between groups. All revisions in both groups were for anterior cruciate ligament graft re‐rupture; none were required for meniscal repair failure.

Abbreviations: ADL, activities of daily living; ADT, anterior drawer test; KOOS, Knee Injury and Osteoarthritis Outcome Score; LMORT, lateral meniscus oblique radial tear; QOL, quality of life.

**Figure 4:**
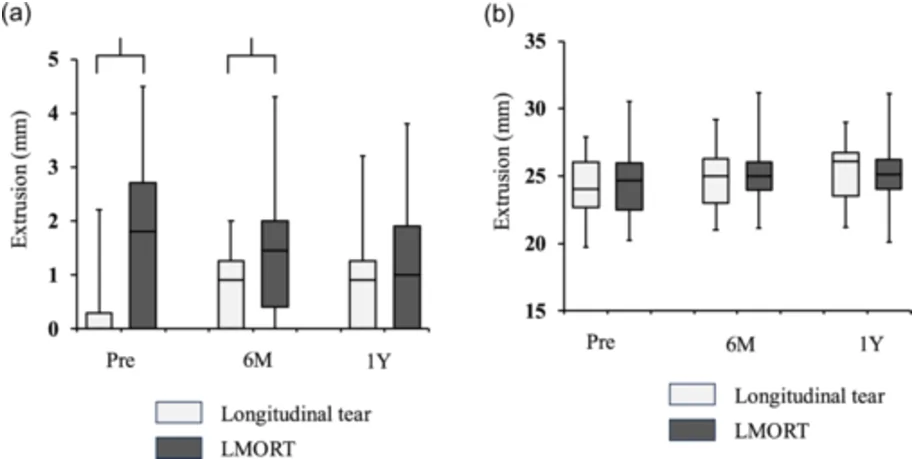
Temporal changes in coronal and sagittal lateral meniscal extrusion. (a) Changes in coronal LME at preoperative, 6‐month and 1‐year assessments in the LMORT and longitudinal tear groups. (b) Changes in sagittal LME at the same assessments. LME, lateral meniscal extrusion; LMORT, lateral meniscus oblique radial tear.

Radiographic joint space width did not differ between the LMORT and longitudinal tear groups at 1 year (5.2 ± 0.8 mm vs. 5.1 ± 0.7 mm, *p* = 0.362) or final follow‐up (5.1 ± 0.8 mm vs. 4.9 ± 0.6 mm, *p* = 0.492) (Table 3). In the LMORT group, mean coronal LME decreased from 1.71 ± 1.31 mm preoperatively to 1.44 ± 1.04 mm at 6 months and 1.14 ± 1.14 mm at 1 year (*p* = 0.010). Conversely, coronal LME in the longitudinal tear group increased from 0.32 ± 0.61 mm preoperatively to 0.71 ± 0.63 mm at 6 months and 0.89 ± 0.98 mm at 1 year (*p* = 0.002). Coronal LME was greater in the LMORT group at baseline (1.71 ± 1.31 mm vs. 0.32 ± 0.61 mm, *p* < 0.001) and 6 months (1.44 ± 1.04 mm vs. 0.71 ± 0.63 mm, *p* = 0.004). Sagittal LME did not differ between groups at any time point. Values in the LMORT group were 24.5 ± 2.7 mm, 25.1 ± 2.1 mm and 25.3 ± 2.4 mm preoperatively, at 6 months, and at 1 year, respectively; corresponding values in the longitudinal tear group were 24.2 ± 2.1 mm, 24.7 ± 2.2 mm and 25.3 ± 2.1 mm (*p* > 0.05) (Figure 4).

**Figure 5:**
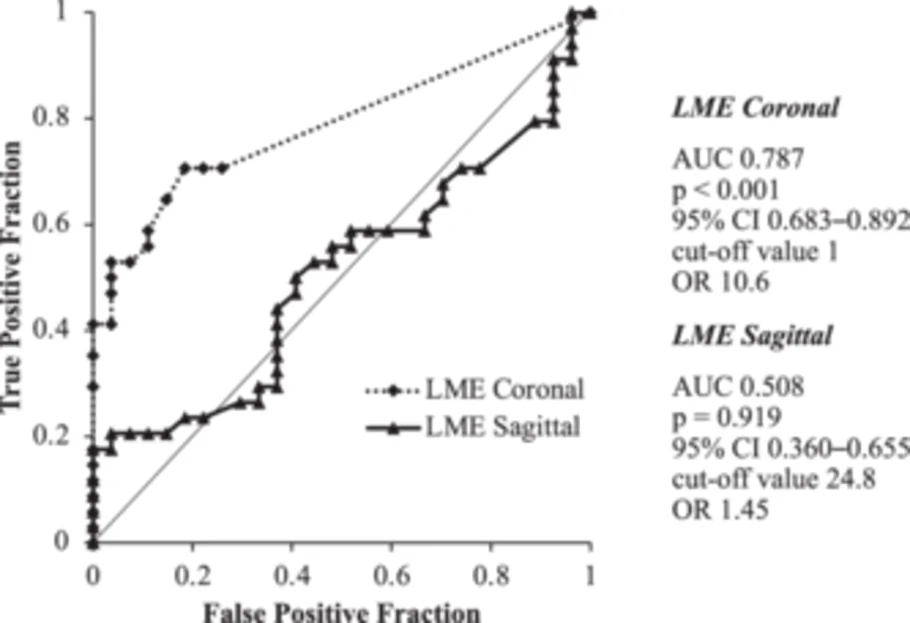
Receiver operating characteristic analysis for distinguishing lateral meniscus oblique radial tear (LMORT) from longitudinal tears. Receiver operating characteristic curves for coronal and sagittal LME. AUC, area under the curve; CI, confidence interval; LME, lateral meniscal extrusion; LMORT, lateral meniscus oblique radial tear.

Multiple linear regression showed that preoperative coronal LME (*β* = −0.514, *p* < 0.001) and number of sutures (*β* = 0.261, *p* = 0.014) were independently associated with the change in coronal LME from preoperative assessment to 1 year. Because change was calculated as the 1‐year minus preoperative value, a negative coefficient indicated greater reduction in extrusion. Thus, greater preoperative coronal LME was associated with greater reduction, whereas more sutures were associated with less reduction (Table 4). Tear pattern (LMORT vs. longitudinal tear) was not associated with changes in coronal (*p* = 0.212) or sagittal (*p* = 0.303) LME, and no examined variable was associated with sagittal LME change (Table 4). ROC analysis showed that preoperative coronal LME had acceptable discriminatory ability for LMORT versus posterior longitudinal tears (AUC = 0.787, *p* < 0.001), based on established criteria defining an AUC of 0.70–0.79 as acceptable [7]. The optimal cutoff was 1.0 mm, yielding an odds ratio of 10.6. Preoperative sagittal LME showed no discriminatory ability (AUC = 0.508, *p* = 0.919) (Figure 5).

**Table 4:** Regression analysis of factors associated with lateral meniscal extrusion (LME).

	Coronal							Sagittal	
	Crude		Adjusted					Crude	
	*β*	*p*‐value	*β*	*p*‐value	95% CI			*β*	*p‐*value
Age	0.083	0.428						−0.029	0.499
BMI	−0.117	0.247						−0.061	0.741
Sex	0.063	0.537						0.442	0.629
Suture number	0.261	0.014	0.261	0.014	0.122	‐	1.038	1.66	0.161
LMORT	−0.149	0.212						−1.202	0.303
Preoperative LME coronal	−0.637	<0.001	−0.514	<0.001	−0.749	‐	−0.338	0.601	0.178
Preoperative LME sagittal	0.037	0.714						−0.174	0.410

*Note*: Independent variables were age, sex, BMI, number of sutures, LMORT and preoperative coronal or sagittal LME. The dependent variable was the change in LME from preoperative assessment to 1‐year postoperative assessment. Adjusted‐model results are presented as β coefficients, *p‐*values, and 95% confidence intervals. BMI, body mass index; CI, confidence interval; LME, lateral meniscal extrusion; LMORT, lateral meniscus oblique radial tear. Variables that did not remain in the adjusted model are presented with crude coefficients only.

## DISCUSSION

The principal finding was that side‐to‐side LMORT repair reduced coronal LME by 1 year despite greater preoperative extrusion than in longitudinal tears. This supports our hypothesis that LMORT repair would reduce extrusion to levels comparable with repaired posterior longitudinal tears while yielding similar clinical outcomes. Because preoperative patient‐reported outcomes were unavailable, these data indicate comparable postoperative outcomes rather than improvement attributable to repair or equivalence between techniques. Additionally, a preoperative coronal LME of >1 mm was associated with LMORT rather than a posterior longitudinal tear. Regression analysis showed that preoperative coronal LME and suture number were associated with postoperative change in extrusion, whereas tear pattern was not. The negative coefficient for preoperative coronal LME indicates that greater baseline extrusion was associated with greater postoperative reduction. Although this may seem counterintuitive because a more extruded meniscus might be harder to reduce, we speculate that traumatic and degenerative extrusion differ mechanistically. In osteoarthritic knees, extrusion develops gradually through attenuation of the meniscotibial and meniscocapsular attachments and intrasubstance meniscal degeneration, potentially fixing the displaced meniscus such that suturing the tear alone may not restore its position. In ACL‐injured knees, by contrast, extrusion may arise acutely from circumferential fibre disruption in meniscal tissue with preserved peripheral attachments and tissue quality. Re‐approximating these fibres may restore hoop function and allow reduction of displacement, unlike chronic degeneration‐related extrusion. This interpretation suggests that reducibility may depend on repair before secondary changes develop and that greater preoperative extrusion in this setting may represent greater correctable displacement rather than a more difficult repair. However, a higher baseline value also allows greater absolute change and may reflect regression to the mean. Consistent with these findings, coronal LME in the LMORT group did not differ from that in the longitudinal tear group at 1 year, and KOOS subscale scores did not differ between groups at 1 year or final follow‐up. Thus, greater preoperative extrusion was not associated with poorer postoperative clinical or functional outcomes. These findings suggest that coronal extrusion associated with LMORT, despite its substantial biomechanical consequences, may be reduced by side‐to‐side repair, whereas sagittal extrusion appears unaffected.

LMORTs are increasingly recognised as clinically important lesions in ACL‐injured knees, with a reported incidence of 7%–14% in primary ACL injuries, although they remain underdiagnosed on MRI [8, 17]. By disrupting circumferential fibres and hoop stress transmission, LMORT increases lateral compartment contact pressure and pivot‐shift‐type instability [21]. Similar biomechanical consequences occur with lateral meniscal posterior root tears, which are also associated with increased meniscal extrusion in ACL‐injured knees [4]. Although meniscal repair during ACLR yields clinical success rates exceeding 85% and healing rates of 70%–85% on MRI or second‐look arthroscopy across tear patterns [6, 9, 24], LMORT‐specific evidence remains limited. Tsujii et al. reported excellent mid‐term outcomes after repair of radial/flap tears of the posterior LM following ACLR, including MRI healing rates of more than 90% and substantial improvements in patient‐reported outcomes [23]. LMORT repairs using side‐to‐side or all‐inside techniques have also achieved healing rates of >70% and favourable clinical outcomes [22]. In the present study, LMORT repairs accounted for 7.4% of primary ACLR procedures and showed reduced coronal meniscal extrusion and clinical outcomes that did not differ from those after posterior longitudinal tear repair.

Increased LME reflects loss of hoop tension and is associated with progressive cartilage degeneration [8, 12]. Minami et al. reported mean coronal LME of 1.3 ± 1.1 mm in patients with lateral meniscal posterior root tears, compared with 0.5 ± 0.5 mm in lateral meniscal tears excluding radial tears [18]. After repair of radial and flap tears of the posterior LM, coronal LME improved by 0.2 mm, whereas sagittal LME worsened by 1.2 mm [23]. The present study showed a similar pattern in the LMORT group: coronal LME was 1.71 mm preoperatively and decreased by approximately 0.6 mm at 1 year, whereas sagittal LME increased by approximately 0.8 mm, consistent with previous findings [23]. Conversely, coronal LME in the longitudinal tear group increased from 0.32 to 0.89 mm, resulting in similar values between groups at 1 year despite opposite trajectories. This finding is consistent with Kashihara et al., who reported that concurrent lateral meniscal repair during ACLR may itself induce LME [14]. One possible mechanism is that vertical all‐inside sutures capture the peripheral meniscal rim and adjacent capsule, potentially drawing the meniscus peripherally during early healing. Because baseline extrusion was minimal (0.32 mm), a small peripheral shift produced a relatively large proportional increase, although the absolute 1‐year value (0.89 mm) remained well below the 4 mm threshold associated with impaired load distribution [5]. Consistent with these imaging findings, KOOS subscale scores in the longitudinal tear group did not differ from those in the LMORT group at either follow‐up. This divergence may reflect the mechanical effect of side‐to‐side repair, which directly re‐approximates disrupted circumferential fibres and may restore hoop function, thereby reducing coronal extrusion, whereas sagittal containment may depend more on capsular and root attachments not addressed by this technique. Three technical factors may contribute. First, the all‐inside device places sutures perpendicular to the tear, apposing the radial edges without fixation between the meniscus and capsule or tibial plateau; therefore, anteroposterior positioning may remain largely unchanged. Second, LMORT spares the root attachment, and anteroposterior position may depend largely on the posterolateral peripheral attachments, the popliteomeniscal fascicles and the meniscofibular and meniscotibial ligaments, rather than the mid‐substance fibres repaired by suturing [10]. Third, inside‐out or outside‐in sutures pass through the capsule and could theoretically provide additional anteroposterior restraint but require a posterolateral approach with potential peroneal nerve exposure. Whether these techniques improve sagittal containment in LMORT remains unknown and warrants further investigation. Clinically, these findings suggest that side‐to‐side repair may reduce coronal extrusion in LMORT, supporting its use for this tear pattern. Furthermore, greater preoperative coronal LME in LMORT than in longitudinal tears suggests that elevated coronal LME on MRI may prompt suspicion of LMORT and careful arthroscopic inspection of the posterior horn. A preoperative coronal LME of >1.0 mm discriminated LMORT from posterior longitudinal tears with acceptable accuracy (AUC = 0.787). This performance is comparable to direct MRI identification of LMORT, which showed 77% sensitivity and 55% specificity [1], suggesting that coronal LME may provide a complementary, readily measurable imaging marker. However, an AUC in this range entails meaningful misclassification [7], so coronal LME alone cannot confirm or exclude LMORT. Instead, elevated preoperative coronal LME should increase suspicion and prompt careful arthroscopic inspection of the posterior horn. Direct tear visualisation and quantitative coronal extrusion provide complementary information from the same MRI examination; when an oblique posterior horn signal is equivocal, coronal LME > 1.0 mm offers an additional reproducible clue to LMORT. Thus, preoperative MRI may identify knees requiring deliberate posterior horn probing rather than confirm the diagnosis. Sagittal LME, however, showed no discriminatory ability (AUC = 0.508). As defined here and by Tsujii et al. [23], sagittal LME represents the anteroposterior meniscal span on the midsagittal slice rather than radial displacement from the tibial margin and may therefore be less sensitive to circumferential fibre disruption. Anteroposterior lateral meniscal position may instead depend largely on posterolateral peripheral attachments. In a robotic cadaveric model, anterior displacement of the posterior horn increased stepwise from 6.3 mm in the intact state to 8.1–8.4 mm after transection of either popliteomeniscal fascicle, 10.2 mm after additional sectioning of the meniscofibular ligament, and 11.4 mm after sectioning of the posterior meniscotibial ligament [10]. Because these structures are not addressed by side‐to‐side repair, this may explain the lack of sagittal response or discrimination between tear patterns. These findings suggest that the coronal plane better reflects the mechanical consequences of LMORT. Accordingly, side‐to‐side repair may improve coronal containment, whereas persistent sagittal LME after repair should not alone be interpreted as repair failure.

Although the absolute changes in meniscal extrusion were small, they may still be clinically relevant. Meniscal extrusion >4 mm has been associated with impaired load distribution and accelerated cartilage degeneration [5]. Postoperative coronal LME in the LMORT group remained well below this threshold and approached that of repaired longitudinal tears. Moreover, the groups showed opposing postoperative trajectories, with decreasing LME in the LMORT group and increasing LME in the longitudinal tear group, suggesting that modest changes may have prognostic relevance. Maintaining LME below clinically meaningful thresholds may therefore be more important for long‐term joint preservation than the absolute magnitude of change.

Patient‐reported outcomes did not differ between groups at 1 year or final follow‐up. At final assessment, all KOOS subscale scores exceeded 85 points, with sports scores of 92.4 in the LMORT group and 94.4 in the longitudinal tear group. Thus, despite greater preoperative extrusion and the biomechanical consequences of LMORT, side‐to‐side repair yielded postoperative symptomatic and functional outcomes similar to those after repair of longitudinal tears. Because preoperative KOOS values were unavailable, these findings demonstrate comparable postoperative outcomes rather than improvement attributable to repair or equivalence between strategies. The absence of between‐group differences should also be interpreted cautiously given the modest sample size and limited power to detect small effects.

This study had some limitations. First, its retrospective, single‐institution design may limit generalisability. Although MRI acquisition was standardised, measurements may have been affected by slice orientation and measurement variability. Second, the modest sample size, particularly in the longitudinal tear group, may have limited detection of small between‐group differences. Third, arthroscopic classification did not assess tissue quality or vascular status, which may influence meniscal healing. Three patients in each group underwent revision surgery for ACL graft re‐rupture, whereas none required revision for meniscal repair failure during a mean follow‐up of 32.5 months. However, the effect of return‐to‐sport timing on graft re‐rupture could not be assessed because actual return‐to‐competition dates were not prospectively recorded. Fourth, residual coronal LME of 1.14 mm remained at 1 year after LMORT repair, and whether adjunctive fixation could further reduce extrusion was not evaluated. Finally, longer follow‐up is needed to determine whether reduced extrusion is associated with better preservation of articular cartilage.

## CONCLUSIONS

Side‐to‐side repair of Types 3 and 4 LMORTs during ACLR reduced coronal LME from 1.71 mm preoperatively to 1.14 mm at 1 year, with clinical outcomes that did not differ from those after longitudinal tear repair. In the multivariate model of coronal LME change from preoperative assessment to 1 year (Table 4), greater preoperative coronal LME was independently associated with greater reduction (*β* = −0.514, *p* < 0.001), whereas more sutures were associated with less reduction (*β* = 0.261, *p* = 0.014). These findings support side‐to‐side LMORT repair as a meniscal preservation strategy associated with reduced coronal extrusion and clinical outcomes that did not differ from those after longitudinal tear repair.

## AUTHOR CONTRIBUTIONS

Kyota Ishibashi participated in the study design, supervised data collection and drafted the manuscript. Hikaru Kristi Ishibashi, Eiji Sasaki and Yuka Kimura assisted with statistical analysis and manuscript drafting and critically revised the manuscript. Yasuyuki Ishibashi conceived and designed the study. All authors have read and approved the final manuscript.

## FUNDING INFORMATION

The authors have no funding to report.

## CONFLICT OF INTEREST STATEMENT

The authors declare no conflicts of interest.

## ETHICS STATEMENT

This retrospective study was approved by the Hirosaki university (Approval No. 2025‐073), and conducted in accordance with the principles of the 1964 Declaration of Helsinki and its later amendments. All participants provided informed consent before enrolment. Permission to reproduce material from other sources: N/A.

## Data Availability

The data that support the findings of this study are available from the corresponding author upon reasonable request.
